# Graph transformer with disease subgraph positional encoding for improved comorbidity prediction

**DOI:** 10.1002/qub2.70008

**Published:** 2025-06-26

**Authors:** Xihan Qin, Li Liao

**Affiliations:** ^1^ Department of Computer and Information Sciences University of Delaware Newark Delaware USA

**Keywords:** comorbidity, graph embedding, graph transformer, human interactome, subgraph positional encoding

## Abstract

Comorbidity, the co‐occurrence of multiple medical conditions in a single patient, profoundly impacts disease management and outcomes. Understanding these complex interconnections is crucial, especially in contexts where comorbidities exacerbate outcomes. Leveraging insights from the human interactome and advancements in graph‐based methodologies, this study introduces transformer with subgraph positional encoding (TSPE) for disease comorbidity prediction. Inspired by biologically supervised embedding, TSPE employs transformer’s attention mechanisms and subgraph positional encoding (SPE) to capture interactions between nodes and disease associations. Our proposed SPE proves more effective than Laplacian positional encoding, as used in Dwivedi et al.’s graph transformer, underscoring the importance of integrating clustering and disease‐specific information for improved predictive accuracy. Evaluated on real clinical benchmark datasets (RR0 and RR1), TSPE demonstrates substantial performance enhancements over the state‐of‐the‐art method, achieving up to 28.24% higher ROC AUC (receiver operating characteristic–area under the curve) and 4.93% higher accuracy. This method shows promise for adaptation to other complex graph‐based tasks and applications. The source code is available at GitHub website (xihan‐qin/TSPE‐GraphTransformer).

## 1 INTRODUCTION

Comorbidity, defined as the simultaneous presence of multiple medical conditions in a single patient [1], significantly influences disease management, therapeutic approaches, and prognostic outcomes [2]. For example, studies have shown that patients with specific comorbidities face various negative outcomes from COVID‐19, highlighting the critical need for thorough comorbidity analysis in managing such pandemics [3, 4, 5]. Grasping the intricacies of comorbidity patterns is crucial for deciphering complex disease interconnections and uncovering common molecular pathways. The network‐based methodologies [6, 7, 8, 9] for predicting and examining these interrelationships significantly enhances our understanding and paves the way for innovative diagnostic and therapeutic strategies.

The human interactome (HI) [10] is a graph/network of collected protein‐protein interactions in human cells. In the HI, nodes represent genes (or their protein products) and edges represent the interactions between proteins. Each node’s connectivity and position within the network encapsulate important information relevant to the corresponding gene’s role in the cellular processes. To extract such information, graph embedding techniques are employed to map nodes to a low‐dimensional continuous vector space, preserving essential graph topology and making it compatible with machine learning models. This embedding representation enables more efficient and accurate analysis, facilitating downstream tasks such as node classification, link prediction, and, in our case, subgraph relationship study for disease comorbidity prediction.

Among the methods leveraging the HI to study disease comorbidity, a recent method, biologically supervised embedding (BSE) [11], significantly outperforms the previous method, geodesic embedding (GE) [12], which uses Isomap [13], by using supervised selection to generate the most biologically relevant embedding vectors. The study of BSE highlights the critical role of disease associations and node connectivity in predicting comorbidities using the HI graph. Building upon these foundational concepts, this study explores the potential of the transformer model to leverage node attention mechanisms and subgraph positional encoding (SPE) for node connectivity and disease associations as emphasized by BSE for comorbidity prediction.

For comorbidity measurement, relative risk (RR) is a widely used metric that quantifies the likelihood of co‐occurrence between studied groups. RR was originally introduced in epidemiological cohort studies, in research demonstrating the link between smoking and lung cancer [14]. RR became a standard in comorbidity analysis following the influential HI study by Menche et al. [10], which showed that disease pairs with RR ≈ 1 exhibit less significant comorbidity, whereas higher RR values indicate stronger associations. Many studies subsequently adopted RR = 1 as a practical threshold for identifying comorbid disease pairs [8, 9, 15]. In earlier state‐of‐the‐art modeling, the GE study [12] explored RR = 0 as a permissive threshold to include weaker disease associations for comorbidity consideration. Building on that, the BSE study [11] adopted both RR = 0 and RR = 1 thresholds to support comprehensive and fair benchmarking.

The transformer model, introduced by Vaswani et al. [16], exerted a profound influence on the development of large language models. Its cornerstone is the multi‐head attention mechanism, comprising both self‐attention for the source language, self‐attention for the target language, and cross‐attention for source–target language relationships. This architecture’s ability to capture diverse aspects of relationships across different attention heads distinguishes it from recurrent neural networks, particularly because of its computational efficiency. Notably, renowned models such as BERT [17] and GPT [18] harnessed the transformer’s architecture to achieve state‐of‐the‐art performance across various tasks. The transformers’ adeptness in capturing contextual information and long‐range dependencies has rendered them a preferred choice not only in language processing but also in various domains including image classification [19, 20, 21], medical data research tasks such as compound–protein interaction prediction [22, 23], and healthcare predictive analyses utilizing electronic health records [24]. Dwivedi et al. introduced a generalized form of the transformer neural network architecture, known as generalized transformer networks (GT) [25], applicable to arbitrary homogeneous graphs, expanding its utility beyond natural language processing (NLP) to graph‐based tasks. This adaptation bridges the gap between the original transformer, which is for sequential data like sentences in large language models (LLMs), and graph neural networks, which operate via message‐passing mechanisms. Notably, their work introduces local attentions among graph nodes and Laplacian eigenvectors as graph nodes’ positional encodings (PEs) in the proposed graph transformer framework.

In this study, we introduced transformer with subgraph positional encoding (TSPE) for disease comorbidity prediction inspired by insights from BSE [11]. TSPE leverages transformer’s attention mechanism to capture node interactions and integrates SPE method for disease association information. Node2Vec was utilized for generating node embeddings. Given the skewness in both benchmark datasets RR0 and RR1, we evaluated TSPE’s performance using ROC AUC (receiver operating characteristic–area under the curve) as the primary metric, which is well‐suited for skewed datasets, and accuracy metrics as a secondary measure. TSPE demonstrated substantial improvements over the state‐of‐the‐art BSE method with support vector machine (SVM) classifier, achieving a 28.24% increase in ROC AUC and a 3.04% increase in accuracy for RR0, with average scores of 0.9489 for ROC AUC and 0.9069 for accuracy. For the RR1 benchmark dataset, TSPE showed a 15.40% increase in ROC AUC and a 4.93% increase in accuracy compared to the state‐of‐the‐art method, achieving scores of 0.8009 for ROC AUC and 0.7294 for accuracy.

## 2 RESULTS

In this section, all results are based on testing the RR0 and RR1 benchmark datasets, which were constructed from the HI and disease comorbidity data introduced by Menche et al. [10] and also used in the BSE study [11], using stratified 10‐fold cross‐validation. Each 10‐fold split of the datasets underwent all designed experimental methods, and the performance metrics were averaged for comparison.

Table 1 compares TSPE against the state‐of‐the‐art method [11] across different scenarios. Firstly, TSPE is compared with the SVM classifier used in Qin and Liao [11] maintaining Node2Vec as the embedding method. Secondly, it is compared with the state‐of‐the‐art BSE method proposed by Qin and Liao [11], with SVM used as the classifier in this comparison, and denoted as BSE_SVM in Table 1. For the RR0 dataset, TSPE outperforms SVM significantly with a 41.80% increase in ROC AUC and a 7.12% increase in accuracy. Compared to BSE with SVM, TSPE shows a 28.24% increase in ROC AUC and a 3.04% increase in accuracy compared to the state‐of‐the‐art method, averaging 0.9489 for ROC AUC and 0.9069 for accuracy. Similarly, for the RR1 benchmark dataset, TSPE shows a 15.40% increase in ROC AUC and a 4.93% increase in accuracy compared to the state‐of‐the‐art method, reaching 0.8009 for ROC AUC and 0.7294 for accuracy.

**TABLE 1:** TSPE against benchmark methods.

Metric	SVM	BSE_SVM	TSPE
RR0			
ROC AUC	0.5309 ± 0.0105	0.6665 ± 0.0301	**0.9489 ± 0.0501**
Accuracy	0.8357 ± 0.0039	0.8765 ± 0.0117	**0.9069 ± 0.0683**
RR1			
ROC AUC	0.5497 ± 0.0079	0.6469 ± 0.0183	**0.8009 ± 0.0152**
Accuracy	0.6150 ± 0.0078	0.6801 ± 0.0166	**0.7294 ± 0.0138**

*Note*: The bold values indicate the model that achieved the best performance on each metric.

Abbreviations: BSE_SVM, biologically supervised embedding_support vector machine; ROC AUC, receiver operating characteristic–area under the curve; RR, relative risk; SVM, support vector machine; TSPE, transformer with subgraph positional encoding.

The RR1 dataset exhibits lower metric scores compared to RR0, suggesting RR1’s potential sensitivity to improvements with more accurate information in various PE methods. We conducted an ablation analysis on PE to assess its impact on increasing metric scores using the RR1 dataset. Table 2 presents the results of the TSPE framework with different PE methods on the RR1 benchmark dataset, measured using stratified 10‐fold cross‐validation.

**TABLE 2:** Performance of transformer models using different positional encoding (PE) methods.

Metric	NoPE	LPE	SPE
10‐fold cross‐validation			
ROC AUC	0.7971 ± 0.0146	0.8007 ± 0.0179	**0.8009 ± 0.0152**
AUPRC	0.8429 ± 0.0168	0.8425 ± 0.0219	**0.8438 ± 0.0199**
Accuracy	0.7214 ± 0.0202	0.7234 ± 0.0202	**0.7294 ± 0.0138**
MCC	0.4340 ± 0.0299	0.4286 ± 0.0423	**0.4578 ± 0.0378**
After removing one outlier			
ROC AUC	0.7951 ± 0.0171	0.7991 ± 0.0198	**0.8010 ± 0.0218**
AUPRC	0.8458 ± 0.0150	0.8454 ± 0.0211	**0.8467 ± 0.0186**
Accuracy	0.7209 ± 0.0177	0.7229 ± 0.0230	**0.7289 ± 0.0203**
MCC	0.4387 ± 0.0275	0.4368 ± 0.0353	**0.4643 ± 0.0337**

*Note*: The comparison is among different positional encoding methods (NoPE, LPE, and SPE). The bold values indicate the best‐performing positional encoding method for each metric.

Abbreviations: AUPRC, area under the precision‐recall curve; LPE, Laplacian positional encoding; MCC, Matthews correlation coefficient; NoPE, absence of PE; ROC AUC, receiver operating characteristic–area under the curve; SPE, subgraph positional encoding.

Both PE methods show improvements in ROC AUC and accuracy compared to using no PE method (NoPE).

The Laplacian positional encoding (LPE) method, introduced by Dwivedi and Bresson [25], enhances ROC AUC by 0.36% and accuracy by 0.20% when integrated into our transformer framework for 10‐fold cross‐validation. After removing one outlier, ROC AUC improves by 0.40% and accuracy by 0.20%. In Dwivedi’s study [25], the focus was solely on accuracy for benchmark datasets (CLUSTER and PATTERN), reporting improvements of 1.03% and 0.859%, respectively. In our case, using LPE as PE shows smaller improvements than those reported in Dwivedi and Bresson [25]. This discrepancy may be due to LPE providing only clustering information in our task, lacking the disease subgraph label information, which is an important factor identified in BSE’s work [11].

However, although LPE achieves slightly higher ROC AUC and accuracy than NoPE, it performs slightly worse in terms of AUPRC (area under the precision–recall curve) [26] and MCC (Matthews correlation coefficient) [27]. This indicates that although LPE helps the model better rank comorbid and non‐comorbid pairs overall (as reflected by ROC AUC), it may not improve, and can even slightly reduce, the model’s confidence in identifying true positives without false positives which is what AUPRC captures. The drop in MCC further suggests that LPE leads to a less balanced classification outcome when evaluated at a fixed threshold.

Our SPE method achieves consistently the highest performance among all PE methods, with a 0.38% increase in ROC AUC and a 0.80% increase in accuracy during 10‐fold cross‐validation, and a 0.59% increase in ROC AUC and 0.80% increase in accuracy after removing one outlier. For AUPRC, SPE slightly but consistently outperforms both LPE and NoPE. This slight improvement, although not as big as that in ROC AUC, is expected, as the RR1 dataset is imbalanced with more positive samples than negative ones. In such a scenario, AUPRC is less sensitive in highlighting improvements when the positive class dominates. Therefore, the consistent gains in AUPRC, although modest, still support SPE’s contribution to improving classification precision. In imbalanced and noisy graph classification tasks, MCC in the 0.3–0.5 range can indicate reasonably good performance depending on task complexity [28, 29]. AUPRC, which better reflects the model’s ability to identify the positive class under class imbalance, complements MCC by emphasizing precision–recall trade‐offs. For MCC, SPE shows statistically significant improvements over both NoPE and LPE. Specifically, during 10‐fold cross‐validation, MCC increases by 2.38% over NoPE (*p* = 4.4474e−04) and by 2.92% over LPE (*p* = 2.2511e−05). After removing one outlier, MCC increases by 2.56% over NoPE (*p* = 5.1374e−04) and by 2.75% over LPE (*p* = 6.0590e−05). These results highlight that incorporating both clustering and local disease label information leads to optimal PE for comorbidity prediction, with SPE consistently outperforming other methods.

## 3 DISCUSSION

In this study, we introduced TSPE for disease comorbidity prediction by leveraging the transformer architecture, traditionally used in NLP tasks, and tailoring it for graph data and subgraph relationship classification. The innovation of TSPE, inspired by BSE’s discoveries, is to utilize node attentions to capture key protein node connections and implement proposed SPE method to incorporate key disease association information for the task.

Our results on real clinical benchmark datasets demonstrate TSPE’s significant superiority over the state‐of‐the‐art BSE method with SVM classifier. By introducing GPE (GEE‐based positional encoding), we effectively reduce the dimensionality of GEE (graph encoder embedding) embeddings from 153 (the number of diseases in the dataset) to a customized dimension. Our proposed SPE integrates LPE and GPE, enabling the model to capture disease associations more effectively at the subgraph level. This integration with node embeddings allows for a comprehensive representation of both node connectivity and disease associations, enhancing the model’s understanding of comorbidity relationships.

Among the PE methods tested, SPE outperformed the popular LPE method, underscoring that integrating clustering and disease information provides the most effective PE strategy for this task. This combined approach harnesses the strengths of both LPE and SPE, effectively capturing essential features for accurate comorbidity prediction.

Regarding limitations, if subgraph labels are unavailable, the proposed SPE cannot be applied. Users may still opt to use TSPE with “NoPE” option or with LPE or propose another PE method to use with our transformer framework. Additionally, although we followed the same data split strategy as the BSE study to ensure a fair comparison, this setup precludes the use of a separate independent test set for evaluating generalization. Future work will explore larger and more up‐to‐date HI datasets constructed from different biological sources to assess the model’s generalization capability beyond a single interactome dataset. We also plan to evaluate generalization within the same interactome by holding out an independent test set. These directions will help determine the robustness of TSPE to variations in interactome structure, biological coverage, and data partitioning.

## 4 CONCLUSION

By integrating both protein node attentions and disease association information, TSPE achieves superior performance in comorbidity prediction compared to the state‐of‐the‐art method using HI data. The ablation analysis on the PE methods for TSPE demonstrated that SPE exhibits the highest efficacy, highlighting the value of combining clustering and disease information. Our findings suggest that the method of integrating PE with node attentions plays a critical role enhancing performance. Given its demonstrated success in predicting comorbidity with disease subgraphs, it is conceivable that TSPE can be adapted for other applications involving various subgraph relationship prediction tasks across different fields.

## 5 MATERIALS AND METHODS

### 5.1 Materials

The HI dataset, along with comorbid disease pairs and their clinically reported RR values, were sourced from the study by Menche et al. [10]. These datasets are also utilized in the BSE study [11]. The RR score in Menche et al. [10] was calculated using a large patient medical history dataset, consisting of records from 13,039,018 patients diagnosed with one or more diseases over a 4‐year period. The HI dataset, curated by domain experts, includes 13,460 protein nodes (denoted by gene IDs coding for proteins) and comprises 153 disease subgraphs. A total of 10,743 disease pairs were used in this study. These pairs were split into two different settings following the methodology of the BSE study [11] for pair comparison. In the first setting, disease pairs with an RR score >0 were marked as positive for comorbidity, and those with an RR score less than or equal to 0 were marked as negative; this dataset is referred to as RR0. In the second setting, disease pairs with an RR score greater than or equal to 1 were marked as positive for comorbidity, and those with an RR score <1 were marked as negative; this dataset is referred to as RR1. In the RR0 dataset, 82.6% of the disease pairs are marked as positive, whereas in the RR1 dataset, 58.4% of the pairs are marked as positive.

### 5.2 Related work

The GT framework [25] proposes two frameworks: one tailored for graphs without edge features, and the other designed for graphs with edge features. Both frameworks employ LPE. In the language transformation task, each vector corresponds to a word token in a sentence. However, unlike the fixed order of words in a sentence resembling a linear graph, the nodes in a graph exhibit arbitrary ordering. Consequently, LPE is employed by directly adding it to the linearly transformed node features to inject graph structural information into the transformer framework. Dwivedi et al.’s ablation analysis reveals that LPE captures superior structural and positional information in comparison to the Weisfeiler–Lehman‐based absolute PE employed in GraphBERT [30].

The LPE process involves two key steps: initially, Laplacian normalization [31] of the graph is conducted, followed by eigen decomposition. Subsequently, the resulting eigenvectors are sorted based on their corresponding nonzero eigenvalues with the *k* smallest eigenvectors chosen to serve as the PE.

(1) L=D−A,

(2) $$\tilde{L}=D^{-\frac{1}{2}}LD^{-\frac{1}{2}}.$$

Equation (1) defines the Laplacian matrix with D being the degree matrix and A being the adjacency matrix. Equation (2) is the normalized Laplacian (denoted as $\tilde{L}$) used by LPE.

(3) $$\tilde{A}=D^{-\frac{1}{2}}AD^{-\frac{1}{2}}.$$

Equation (3) is the definition of normalized adjacency matrix ($\tilde{A}$), where D is the degree matrix, A is the unnormalized adjacency matrix.

(4) $$\tilde{L}=I-\tilde{A}.$$

By substituting Equations (1 and 3) into Equation (2), Equation (4) is derived which is used to calculate normalized Laplacian matrix ($\tilde{L}$).

(5) $$\tilde{L}=U\Lambda U^{T}.$$

Equation (5) represents the eigen decomposition of the normalized Laplacian matrix ($\tilde{L}$). In these equations, U is an “n×n” matrix comprising orthonormal eigenvectors ($u_{1}$, $u_{2}$, …, $u_{n}$), and Λ is an “n×n” diagonal matrix consisting of eigenvalues ($\lambda_{1}$, $\lambda_{2}$, …, $\lambda_{n}$). Following this, the k eigenvectors corresponding to the k smallest eigenvalues are selected for LPE encoding with the value of k determined by the user.

Another crucial aspect of the GT framework [25] is its consideration of graph sparsity. Given that real‐world graphs are typically large and sparse, with numerous nodes but limited connectivity between them, Dwivedi et al. proposed focusing attention on locally connected nodes rather than performing full attention across all nodes which is the attention mechanism used in language transformer models. Their results demonstrate that this localized attention mechanism surpasses two baseline models: GCN [32] and GAT [33]. Whereas GAT employs attention on local neighborhood connections without considering the entire graph structure, GCN utilizes convolutional operations over the entire graph to aggregate information from neighboring nodes. Consequently, GT’s focus on neighborhood attention, coupled with LPE for graph structure information, bridges the gap between these two approaches, outperforming both.

TransformerGO [34], introduced by Ieremie et al., applies the transformer framework to predict protein‐protein interactions (PPIs) by capturing the semantic similarity between sets of gene ontology (GO) terms represented as a graph. Unlike the language transformer framework, TransformerGO employs unmasked multi‐head attention in the decoder, enabling the model to attend to subsequent positions in the GO term node sets for specific proteins. This attention mechanism is crucial for accurately predicting the relationships between proteins, distinguishing between positive and negative interactions across datasets from *Saccharomyces cerevisiae* and *Homo sapiens*. TransformerGO outperforms classic semantic similarity measures and state‐of‐the‐art machine‐learning‐based approaches in PPI prediction.

Node2Vec is utilized in TransformerGO [34] to process graph data by transforming each protein’s annotated GO terms into embedding vectors which serve as input for TransformerGO. Node2Vec [35] is a widely used embedding method that leverages biased random walks to simulate both depth‐first search and breadth‐first search strategies. These random walks are guided by the return parameter (*p*) and the in‐out parameter (*q*), which control the likelihood of revisiting nodes and exploring new neighborhoods, respectively. A higher value of *p* encourages the walk to remain close to the starting node, promoting DFS‐like local exploration. Conversely, a lower value of *q* favors exploration of nodes further from the starting point, promoting BFS‐like global exploration. The resulting random walks are then fed into a skip‐gram model to produce embeddings, where nodes within a specified window size have closer embedding values.

### 5.3 Methods

**FIGURE 1:**
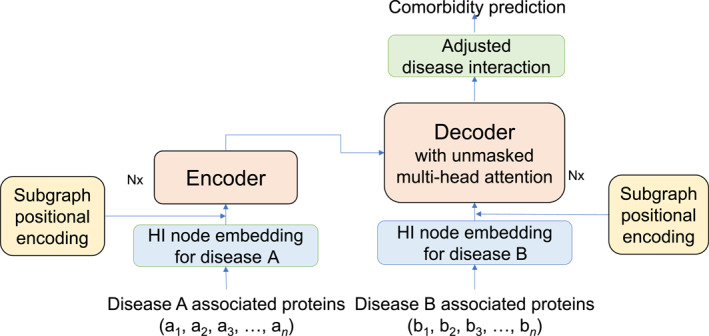
Transformer with subgraph positional encoding.

Drawing inspiration from the advancements made by BSE [11], TSPE is proposed with an architecture illustrated in Figure 1. Our objective is to predict the relationship (whether comorbid or not) between two diseases, each is a subgraph in the HI, comprising a set of protein nodes associated with the corresponding disease. In this framework, understanding the connections between nodes within subgraphs is crucial for predicting disease subgraph relationships. Similar to how a language transformer model learns from the attention between words to comprehend sentences, the proposed framework learns from the attention between nodes to understand and predict relationships between subgraphs. However, unlike words in a sentence, nodes within subgraphs lack a fixed order. Therefore, we propose using SPE to inject each node’s subgraph information, rather than a fixed order, into the framework.

As this task involves classifying the relationship between any two given disease subgraphs, rather than “translating” one subgraph into another, inspired by TransformerGO [34], the unmasked multi‐head attention mechanism is employed in both the encoder and decoder. This ensures that the self‐attention in decoder can learn to attend to all nodes within the subgraph, just as the encoder does. The output from the decoder maintains the same dimensions as the decoder’s input. Each vector represents the interactions of proteins in disease B with every other protein in disease A, for a given disease pair (A and B). Because this is a binary classification task, the decoder’s output is adjusted for binary classification adopting the idea from TransformerCPI [36]. The equations below describe these processes.

(6) $$s=\text{softmax}\left({\left\|X\right\|}_{2,\text{col}}^{2}\right),$$

(7) $${\left\|X\right\|}_{2,\text{col}}^{2}=\left[{\left\|X_{:,1}\right\|}_{2}^{2},{\left\|X_{:,2}\right\|}_{2}^{2},\ldots ,{\left\|X_{:,n}\right\|}_{2}^{2}\right].$$

Given an interaction matrix *X*, with dimensions *m* × *n*, which is the output from the decoder. A scoring vector *s* is first calculated using Equation (6). The expression ${\left\|X\right\|}_{2,\text{col}}^{2}$ denotes the square of column‐wise L2 norm, resulting in a vector of size 1 × *n*, where *n* denotes the number of columns. Here, the L2 norm is calculated as the square root of the sum of the squares of its components applied to the column vectors. This can be expressed in Equation (7), where ${\left\|X_{:,j}\right\|}_{2}^{2}$ represents the square of the L2 norm of the *j*th column. Subsequently, the softmax function is applied to normalize the values within the vector, ensuring that they sum up to 1. As a result, the scoring vector *s* has dimensions 1 × *n*.

(8) $${\hat{y}}^{\prime }=\sum_{j=1}^{n}{\left(X\text{diag}\left(s\right)\right)}_{.j}.$$

As shown in Equation (8), the scoring vector *s* is then used to weight each column in matrix *X*. Given *s* with elements $\left[s_{1},s_{2},\text{…},s_{n}\;\right]$ and size 1 × *n*, where each $s_{i}$ serves as a weight, and *X* $\left[X_{1},X_{2},\text{…},X_{n}\;\right]$ with size *m* × *n*, where each $X_{i}$ is a column of *X*, the operation Xdiag(s) results in $\left[{s_{1}X}_{1},{s_{2}X}_{2},\text{…},{s_{n}X}_{n}\;\right]$ with a size of *m* × *n*. The candidate ${\hat{y}}^{\prime }$ is then obtained by summing along *n* columns of the resulting matrix, yielding a vector of dimension *m* × 1. ${\left(X\text{diag}\left(s\right)\right)}_{.j}$ represents the column‐wise sum of the product of Xdiag(s).

(9) $$\hat{y}=\sigma \left({W\hat{y}}^{\prime }+b\right),$$

(10) $$L_{\text{BCE}}\;\left(\hat{y},y\right)=-\frac{1}{N}\sum_{i=1}^{N}\left(y_{i}\;\log\hat{y}+\left(1-y_{i}\right)\log\left(1-\hat{y}\right)\right).$$

The candidate ${\hat{y}}^{\prime }$ is then passed through a linear layer followed by the sigmoid activation function to compute the prediction $\hat{y}$. Subsequently, the binary cross entropy loss (BCELoss) is employed to compare the result with the ground truth, yielding the loss for training. The sigmoid layer and BCELoss are implemented together in PyTorch as a single class called BCEWithLogitsLoss [37].

In the HI dataset, neither node features nor edge features are available. Therefore, for TSPE, the input for each disease subgraph is obtained by first generating the graph embedding and then selecting the corresponding protein embedding vectors for each specific disease subgraph. Node2Vec [35] is employed to generate the node embeddings. We applied the Python Node2Vec package using their default setting with *p* = 1 and *q* = 1, so that the random walk behaves neutrally, resembling a standard random walk without a bias toward either local or global exploration. This balanced approach captures a mix of structural features but without a strong emphasis on either DFS‐like or BFS‐like behavior. And we set the window size to 2 to focus on capturing local relationships.

**FIGURE 2:**
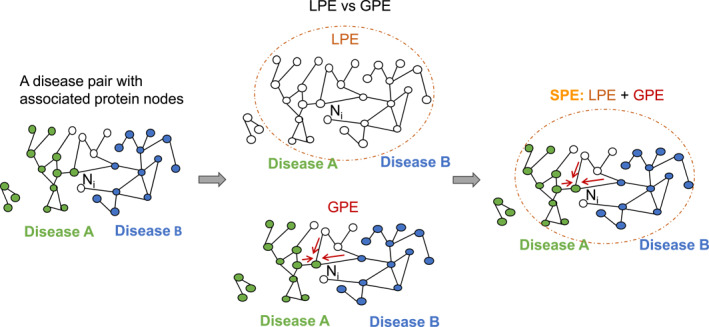
SPE methods for node *N*_*i*_. LPE reaches out to all nodes in a single connected component, enclosed in the dashed circle, indiscriminate of the nodes’ association with disease types. In contrast, GPE incorporates the nodes’ disease associations, which are color‐coded green and blue, and places greater emphasis on neighboring nodes as indicated by the red arrows. SPE combines both LPE and GPE, not only captures further connections of the node but also incorporating the disease information gathered from the neighborhood. GPE, graph encoder embedding‐based positional encoding; LPE, Laplacian positional encoding; SPE, subgraph positional encoding.

The BSE study [11] underscored the significance of both disease association and node connectivity for comorbidity prediction. In our proposed framework, the node attention mechanism is used to capture node connectivity information. Given the absence of node features in the HI dataset, our node embeddings focus on representing the graph structure with an emphasis on local node connectivity. However, this approach lacks information about disease associations, specifically how gene nodes are categorized into different disease subgraphs. To address this issue, we propose adding disease subgraph information as PE as shown in Figure 2.

The LPE in the popular GT framework [25] selects eigenvectors associated with the smallest non‐zero eigenvalues. These smallest eigenvalues are typically used for graph partitioning to identify clusters or communities within the graph, thereby incorporating cluster or subgraph information into the PE. The authors of LPE attribute GT’s success to this feature as it outperformed the baseline models GCN [32] and GAT [33]. As illustrated in Figure 2, although LPE can capture clustering information, it still lacks the ability to incorporate disease associations (i.e., the disease labels of each node). To address this limitation, we propose a novel approach, GPE, based on GEE [38], to incorporate disease association.

GEE [38], proposed by Shen et al., shares similar properties with spectral embedding and is known for its rapid processing capabilities, capable of handling billions of edges within minutes on a standard PC [39]. GEE embedding *Z* is generated by a weight matrix, *W*, derived from known subgraph information and multiplied by either the adjacency matrix, diagonal augmented adjacency matrix, or Laplacian normalization matrix. The primary advantage of GEE lies in its utilization of the weight matrix *W*, which is directly based on subgraph information, providing more disease association information compared to Laplacian eigenvectors used in LPE [25]. Moreover, GEE is much faster to perform than the Laplacian eigenvectors. The GEE embedding matrix, *Z*, constructed by the adjacency matrix, is defined as follows:

(11) Z=AW,

where *A* represents the adjacency matrix and *W* denotes the weight matrix. The size of the *W* matrix is *N* × *K*, with *N* representing the number of nodes and *K* indicating the number of label types. In our dataset, *K* is the total number of disease types which is 153 diseases. For a node *i* belonging to disease subgraph *j*, $W_{ij}$ is calculated as 1/$n_{j}$, where $n_{j}$ represents the number of nodes within disease subgraph *j*.

The challenge associated with using GEE stems from the size of *K*. There are two main reasons for this: firstly, if there are numerous subgraphs, *K* can become exceedingly large; secondly, when employing GPE for summation with input node embedding, ensuring both embeddings possess the same dimensionality can be problematic, as they typically differ. To address this issue, we propose GPE which is outlined below.

(12) $$Z=U\Sigma V^{T}.$$

First, perform the singular value decomposition of *Z*. In Equation (12), *U* represents the left singular vectors, *Σ* is a diagonal matrix with singular values on the diagonal, and *V* represents the right singular vectors.

(13) $$\text{GPE}=U_{d}.$$

Then, as shown in Equation (13), select the first *d* largest left singular vectors based on the singular values to constitute the embedding. The value of *d* is determined by the user.

Next, we propose subgraph positional encoding (PSE) to integrate both the clustering information from LPE and the local disease label information from GPE. As depicted in Equation (14), for a node embedding matrix *M* (shown in the blue box in Figure 1), GPE incorporates PE by first adding LPE to *M*, and then concatenates with GPE to generate the final embedding *E* which serves as the input to the encoder and decoder. This concatenation method enables flexible weighting of LPE and GPE contributions.

(14) E=[(M+LPE),GPE].

Overall, our TSPE model design (Figure 1) utilizes the novel SPE (Figure 2) to encode key disease subgraph and clustering information from the graph into embedding representations. For each disease pair, the embeddings of nodes belonging to different diseases are input separately into the transformer framework’s encoder and decoder. Through the attention mechanism, TSPE emphasizes key node connectivity, which is particularly valuable for revealing previously unknown connections absent in the HI data (currently estimated to be only 20% complete [10]) for the task. This approach highlights relationships within and between disease node sets, enabling effective comorbidity prediction on the test set.

We evaluate the performance of TSPE against the state‐of‐the‐art BSE method [11], which uses a SVM classifier, for comorbidity prediction. Because of the need for a fair and consistent comparison with BSE, we restrict our primary evaluation to the same metrics reported in their work. Given the skewness in both benchmark datasets, RR0 (82.6% positive comorbid pairs) and RR1 (58.4% positive pairs), we prioritize reliable metrics: ROC AUC as the primary metric and accuracy as the secondary metric. We selected ROC AUC as the primary metric because it is a threshold‐independent metric that assesses the model’s ability to rank positive (comorbid) and negative (non‐comorbid) disease pairs across a range of decision boundaries. This is especially valuable when evaluating architectural improvements in the model, rather than optimizing for a specific classification threshold. ROC AUC summarizes the trade‐off between the true positive rate and false positive rate at all possible thresholds, providing a robust measure of discriminative ability.

To provide a deeper assessment of model quality, we additionally perform an ablation analysis comparing TSPE to the same transformer framework without SPE (NoPE) and with LPE only. In this analysis, we incorporate two additional metrics: AUPRC [26] and MCC [27]. AUPRC reflects the model’s ability to retrieve true positives while minimizing false positives, providing insight into model precision in imbalanced settings [26]. MCC captures the balance across all four confusion matrix elements (true positive, true negative, false positive, and false negative), accounting for both types of errors, and is widely considered appropriate for imbalanced binary classification [40]. For MCC computation, the classification threshold is determined using Youden’s *J* index [41], a principled criterion that maximizes the sum of sensitivity and specificity (*J* = sensitivity + specificity − 1), thereby ensuring a fair and objective decision boundary across models. Detailed hyperparameters and settings for TSPE used in the experiments are provided in Table 3.

**TABLE 3:** TSPE parameters.

Layers	3
Learning rate	1e‐04
Batch size	20
Valid	0.1
Dropout	0.2
Node embedding dimension	64
Number of heads	8
Position encoding dimension	GPE: 8
	LPE: 64

Abbreviations: GPE, graph encoder embedding‐based positional encoding; LPE, Laplacian positional encoding; TSPE, transformer with subgraph positional encoding.

## AUTHOR CONTRIBUTIONS

**Xihan Qin**: Conceptualization; formal analysis; investigation; methodology; project administration; resources; supervision; validation; visualization; writing—original draft; writing—review and editing. **Li Liao**: Investigation; supervision; validation; writing—review and editing.

## CONFLICT OF INTEREST STATEMENT

The authors declare no conflicts of interest.

## ETHICS STATEMENT

This study does not involve human participants, animal research, or the use of personally identifiable data.

## Data Availability

The comorbidity data used in this study was provided by Dr. Joerg Menche. A test case and the code for our proposed model are available at the GitHub website (xihan‐qin/TSPEGraphTransformer).
